# Public remotely sensed data raise concerns about history of failed Jagersfontein dam

**DOI:** 10.1038/s41598-023-31633-5

**Published:** 2023-04-05

**Authors:** Luis Alberto Torres-Cruz, Christopher O’Donovan

**Affiliations:** School of Civil and Environmental Engineering, University of the Witwatersrand, Johannesburg, South Africa

**Keywords:** Civil engineering, Natural hazards, Environmental impact

## Abstract

A mine waste deposit known as a tailings dam recently failed in the town of Jagersfontein, South Africa. The failure occurred amidst global concern about the safety record of these structures. Herein we use public remotely sensed data to gain insights into the construction history of the dam. The data suggest a construction sequence that is inconsistent with sound tailings management practices: asymmetric deposition, erosion gullies, large ponds and absence of beaches. These observations highlight the criticality of adhering to good construction practices and the potential of public data to monitor such adherence. Additionally, we present commercially available very high resolution satellite images to illustrate some of the immediate consequences of the failure.

## Introduction

**Figure 1:**
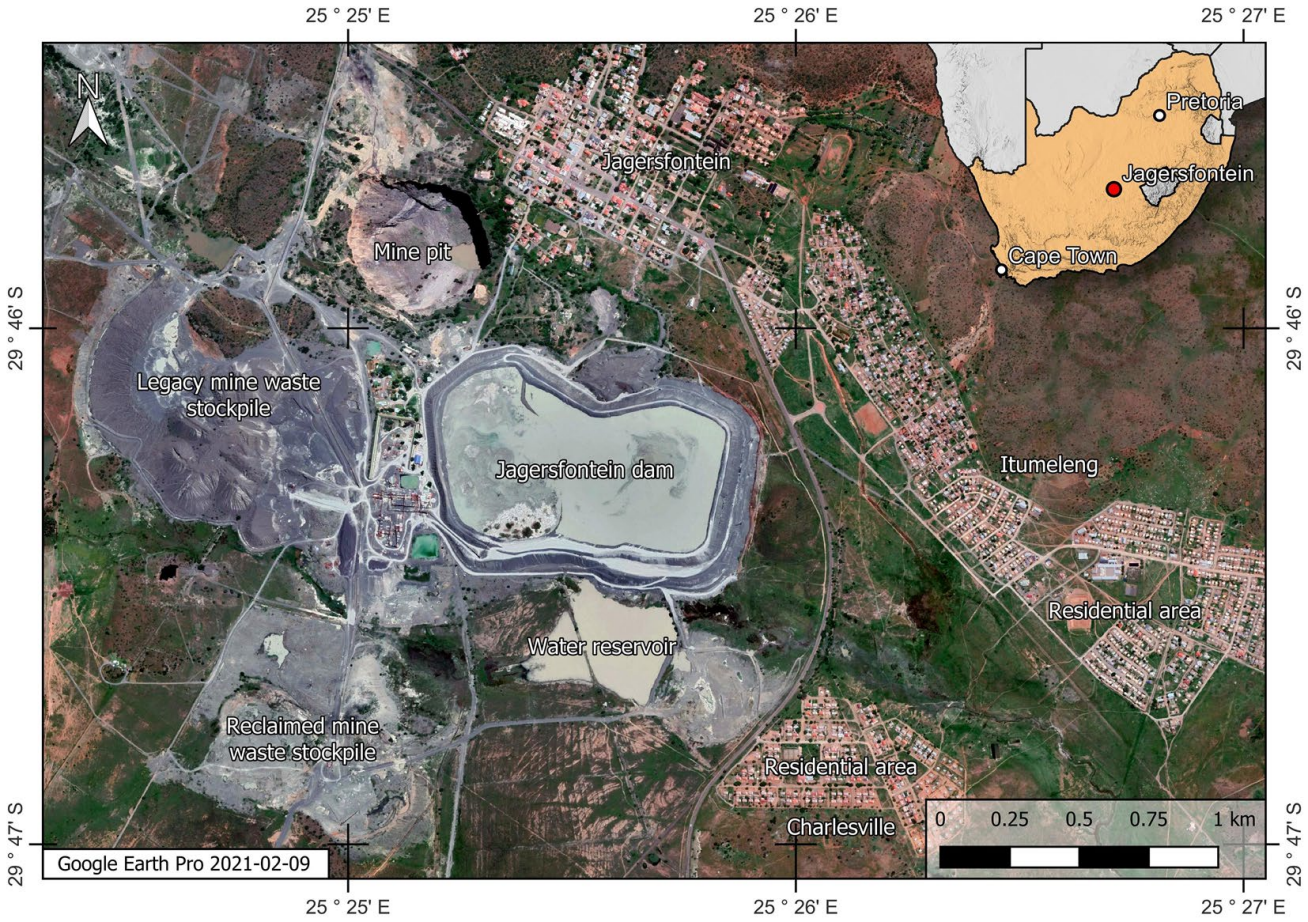
The Jagersfontein dam and its surroundings 19 months before failure, created using QGIS software version 3.26 (http://www.QGIS.org).

On Sunday 11 September, 2022 a diamond mine waste storage facility, known as a tailings dam, failed in the town of Jagersfontein, South Africa^1^. The failure released a mudflow that killed at least one person and destroyed multiple houses. Figure 1 presents the immediate surroundings of the tailings dam prior to failure and highlights the proximity of residential areas to the dam. The tragic failure occurred at a time of heightened global concern around the safety of tailings dams, especially after a failure in Brumadinho, Brazil killed 270 people in 2019^2–4^. The Brumadinho failure prompted the creation of the Global Industry Standard for Tailings Management which strives to achieve "zero harm to people and the environment"^5^. The Jagersfontein failure was a reminder that there is still much work to do to achieve this objective. Herein we use the power of optical remotely sensed data to gain insights into the construction history of the Jagersfontein dam, precursors of failure and the immediate failure consequences.

Mining activities in Jagersfontein date back to the 1870s. The mine was initially operated as an open pit and changed to underground mining in 1913. The De Beers Group acquired the mine in 1931 and operated it until 1971 when underground ore extraction ceased. The De Beers Group sold the mine in 2010 and subsequent owners reprocessed the legacy mine waste already on surface without further ore extraction. At least two more ownership changes happened after 2010 including one that took place only months before the failure of the dam. At the time of the failure, and during most of the post-2010 period, the mine was owned by the subsidiary company Jagersfontein Developments^6–8^.

Unlike conventional water retention dams, tailings dams are often constructed progressively over their entire operational life which can easily span decades^9,10^. This poses the double challenge of ensuring that high construction quality standards are maintained over a long period of time and that the construction process is duly documented^11^. Optical remotely sensed data are valuable for monitoring certain aspects of the operation and to investigate the construction history^12–14^. For instance, investigations into the failure of the Fundão and Brumadinho tailings dams in Brazil used public and commercial optical satellite images to investigate their construction and operation histories^13,14^. Herein we use optical satellite imagery in a similar manner to investigate the history of the Jagersfontein dam and some of the immediate consequences of the failure. Importantly, our investigation into the pre-failure history of the dam relies exclusively on open access data. As such, our work contributes to empower a wide variety of stakeholders interested in proactively monitoring the safety of tailings dams. We also employed commercial satellite imagery although only to assess the consequences of failure.

Because we have not accessed the site, our observations have not been validated by ground truth. However, they illustrate situations that should trigger site inspections by those tasked with ensuring the stability of these structures. Our analysis also informs hypotheses that can be further explored during an in situ investigation into the causes of failure^15^.

## Results

### Construction and operation history of the Jagersfontein dam after 2010

**Figure 2:**
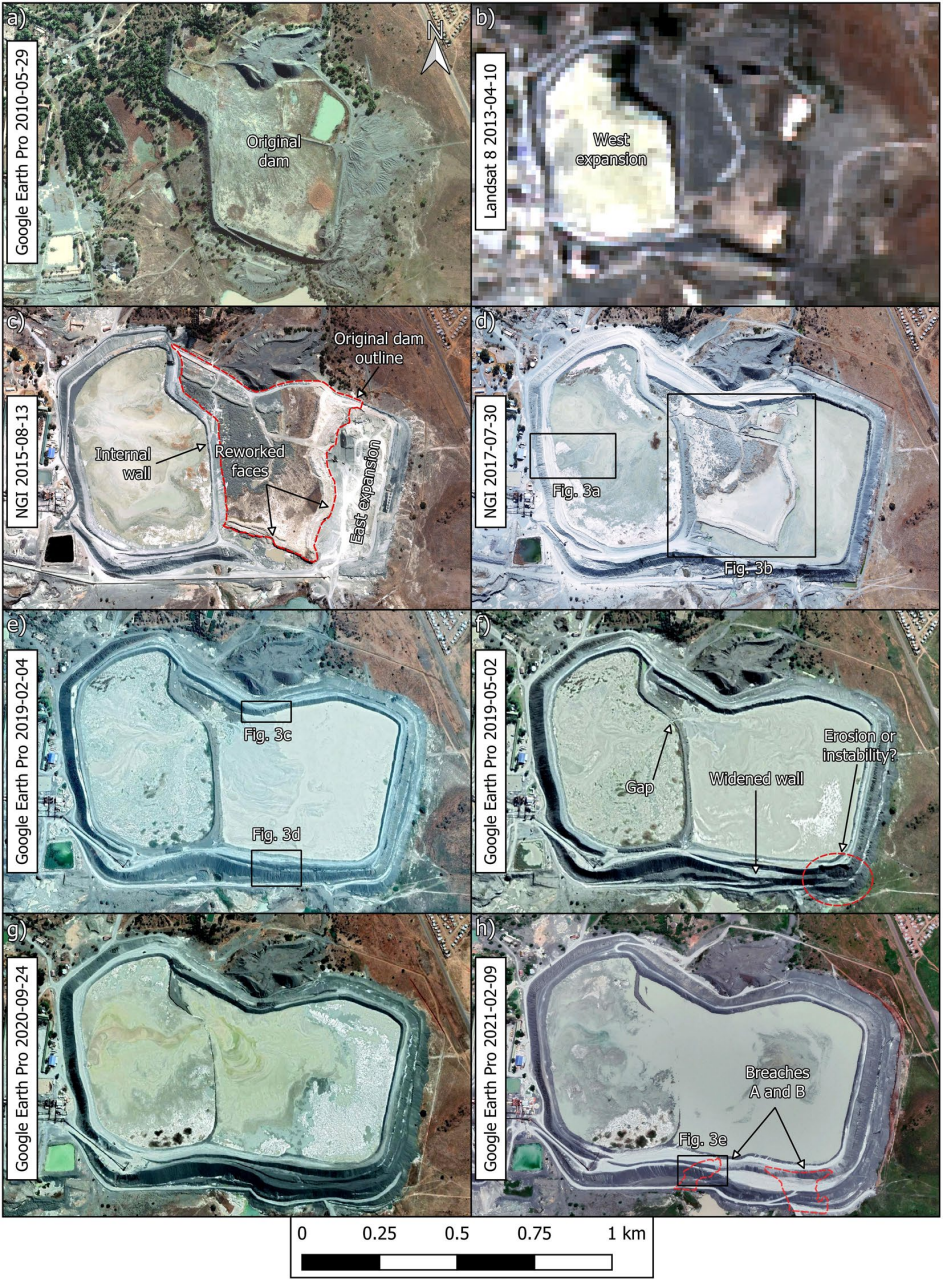
Historical imagery of the Jagersfontein dam. (**a**) Configuration prior to reprocessing of legacy mine waste. (**b**) West expansion is visible by April 2013. (**c**) Deposition in the west expansion concurrent with reworking of the original dam and activity in the east expansion. (**d**) Furrows are visible by July 2017. (**e**–**g**) Eastward migration of tailings from the west expansion through a gap in the internal wall in 2019 and 2020. (**h**) Internal wall is almost entirely buried. NB: Part (**h**) shows location of breaches A and B which developed during failure (Fig. 5). Created using QGIS software version 3.26 (http://www.QGIS.org).

**Figure 3:**
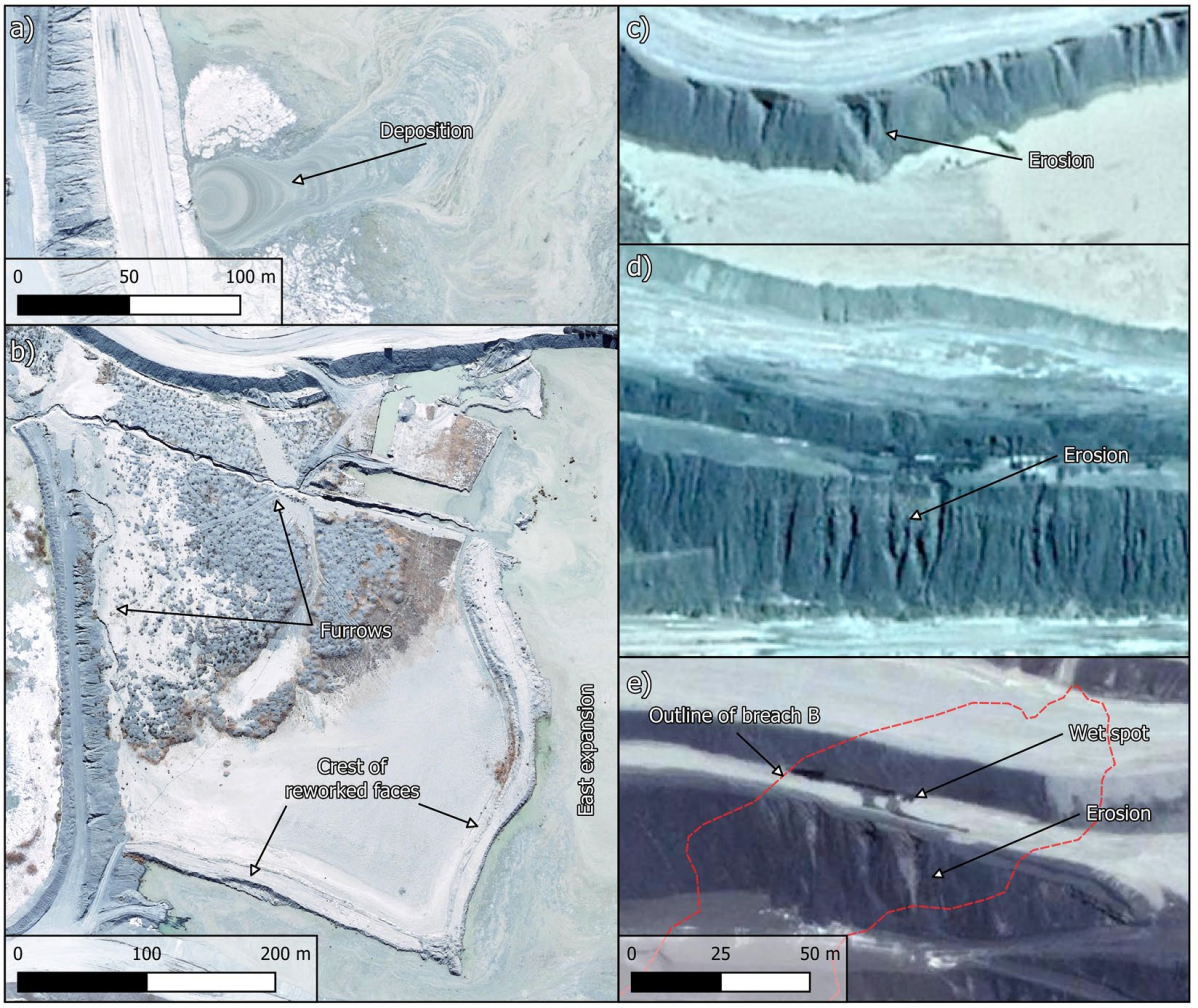
Details of the dam's history. (**a**) Deposition into west expansion enlarged from Fig. 2d. (**b**) Furrows in original dam enlarged from Fig. 2d. (**c** and **d**) Erosion gullies enlarged from Fig. 2e. (**e**) Wet spot and erosion gullies enlarged from Fig. 2h with outline of breach B which developed during failure (Fig. 5). NB: Parts (**c**), (**d**) and (**e**) share the same scale. Created using QGIS software version 3.26 (http://www.QGIS.org).

Aerial and satellite images suggest that the Jagersfontein tailings dam and nearby mine waste stockpiles did not undergo significant changes between mine closure in 1971 and the onset of surface mine waste reprocessing in 2010 (Fig. S1). Accordingly, the focus herein is on the post-2010 development of the dam (Fig. 2). Hereafter we use the phrase 'original dam' to refer to the 2010 configuration of the dam prior to the deposition of tailings resulting from waste reprocessing (Fig. 2a). The images show that by April 2013, the original dam had almost doubled its footprint due to an expansion built on its west side (Fig. 2b). By August 2015, all tailings were being deposited in the west expansion while parts of the original dam were being reworked and activity in the area that would become an east expansion was also underway (Fig. 2c). By July 2017, tailings were being initially deposited into the west expansion and subsequently transported to the original dam and east expansion via two furrows connected to a gap in the internal wall (Figs. 2d, 3a,b). By February 2019, erosion gullies 4 to 5 m wide had developed on the internal face of the northern wall and on the external face of the portion of the southeastern wall that eventually failed (Figs. 2e, 3c,d). By May 2019, the furrows were almost entirely buried but eastward migration of tailings deposited in the west expansion continued through the gap in the internal wall (Fig. 2f). The increased width of the southeastern wall, which eventually failed, may indicate that buttressing efforts were underway. The southeastern corner of the dam exhibited an irregular geometry which may have been due to erosion or slope instability^16^. Eastward migration of tailings continued during 2020 (Fig. 2g) and by February 2021, the internal wall had been almost entirely covered and the southeastern wall remained significantly wider (potentially buttressed) than the remainder of the perimeter wall (Fig. 2h). A segment of the perimeter wall appeared wet and the slope directly underneath it exhibited erosion gullies (Figs. 2h and 3e). A preliminary stereoscopic analysis of satellite images indicates that the dam grew by ~ 18 million m^3^ to accommodate the tailings that resulted from the post-2010 reprocessing of legacy mine waste^17^.

**Figure 4:**
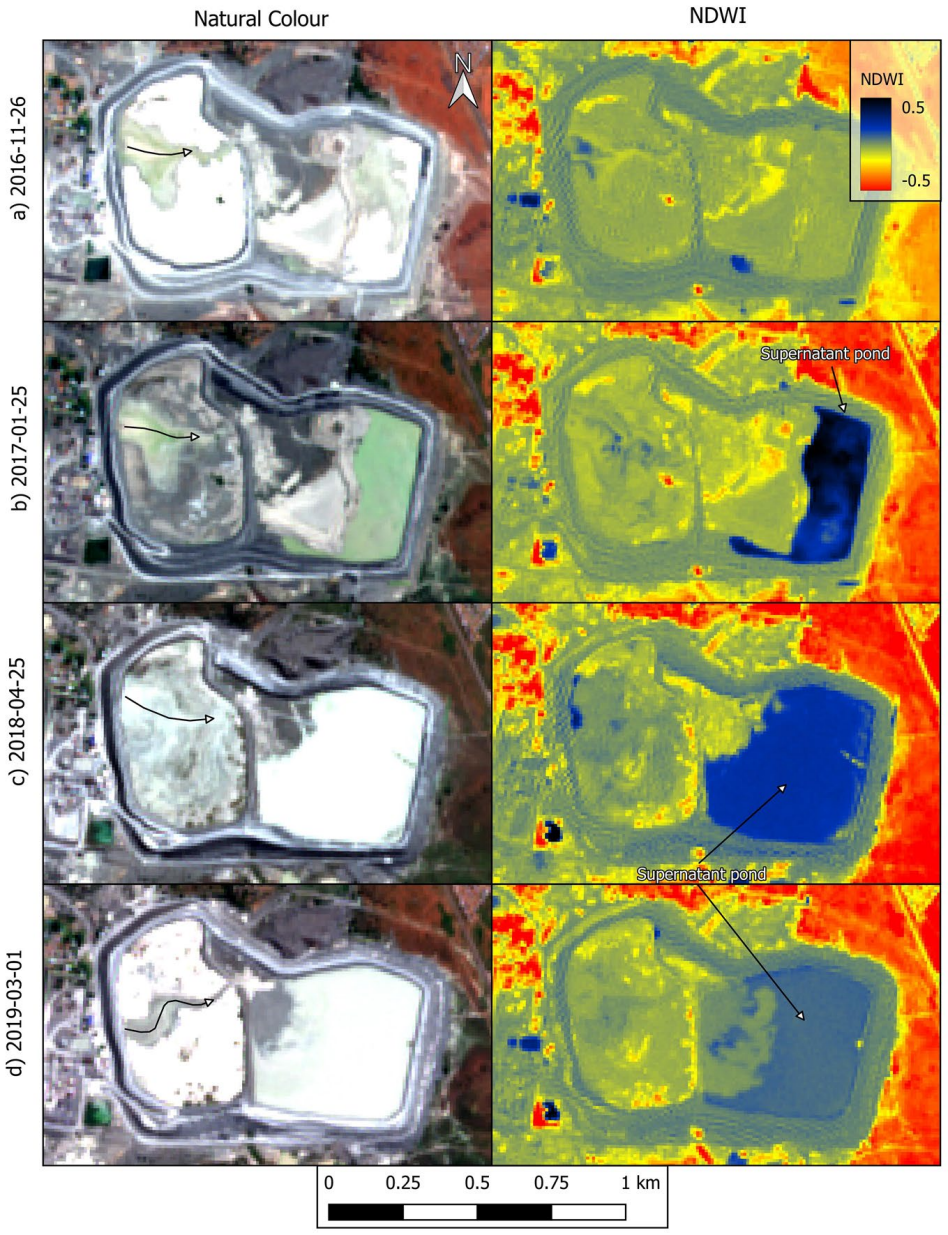

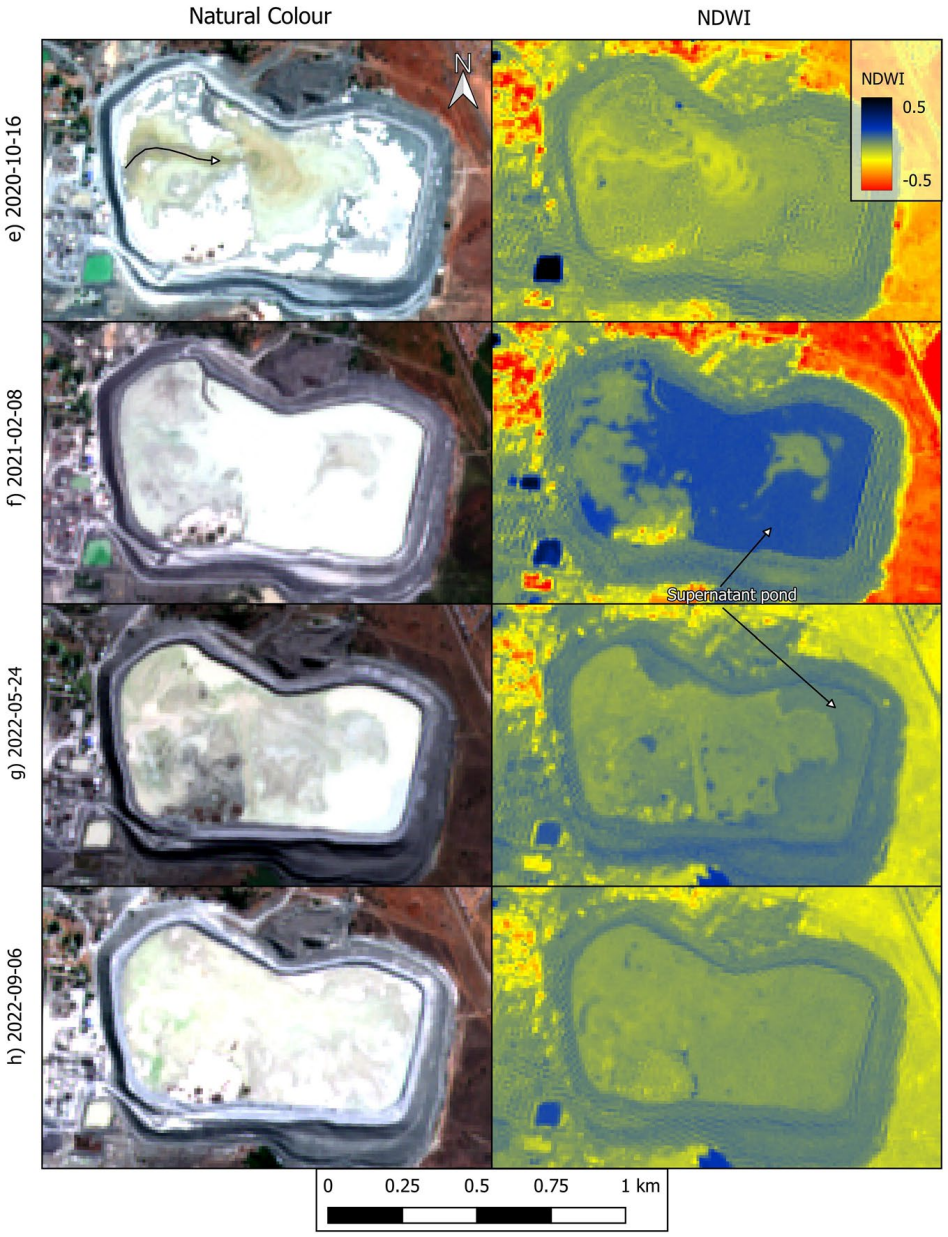
Natural colour and NDWI renderings of Sentinel-2 images captured from 2016 to 2022. Natural colour renditions of parts (**a**) to (**d**) show eastward migration of tailings after initial deposition in the west expansion. NDWI renditions of parts (**b**) to (**d**) show a large supernatant pond located against the perimeter wall. Natural colour rendition of part (**e**) shows eastward migration of tailings after initial deposition in the west expansion. NDWI renditions of parts (**f**) and (**g**) show a large supernatant pond located against the perimeter wall. There is no discernible supernatant pond in part (**h**), captured 5 days before failure. NB: curved arrows indicate the inferred eastward migration of tailings after deposition. Created using QGIS software version 3.26 (http://www.QGIS.org).

The natural colour and normalised difference water index (NDWI)^18^ images in Fig. 4 indicate several instances in which significant amounts of supernatant water accumulated atop the dam, especially over the east expansion and original dam (see details in figure caption). Interestingly, Fig. 4h, captured only 5 days before the failure, suggests that there was no significant pond of supernatant water atop the dam. In fact, although Sentinel-2 images are captured every five days^23^ and clouds did not often compromise the images, one has to go as far back as 24 May 2022 (more than three months before failure) to find an image in which an extensive pond can be identified (Fig. 4g). This suggests that while large decant ponds were present in the historical development of the dam, they were not present in the months immediately prior to the failure. Several images in Fig. 4 (see details in figure caption) also indicate that tailings deposition was taking place predominantly, perhaps exclusively, from the west wall of the dam. This is consistent with the pattern of deposition observed in the higher resolution images (Figs. 2d–g and 3a).

### The immediate consequences of failure

**Figure 5:**
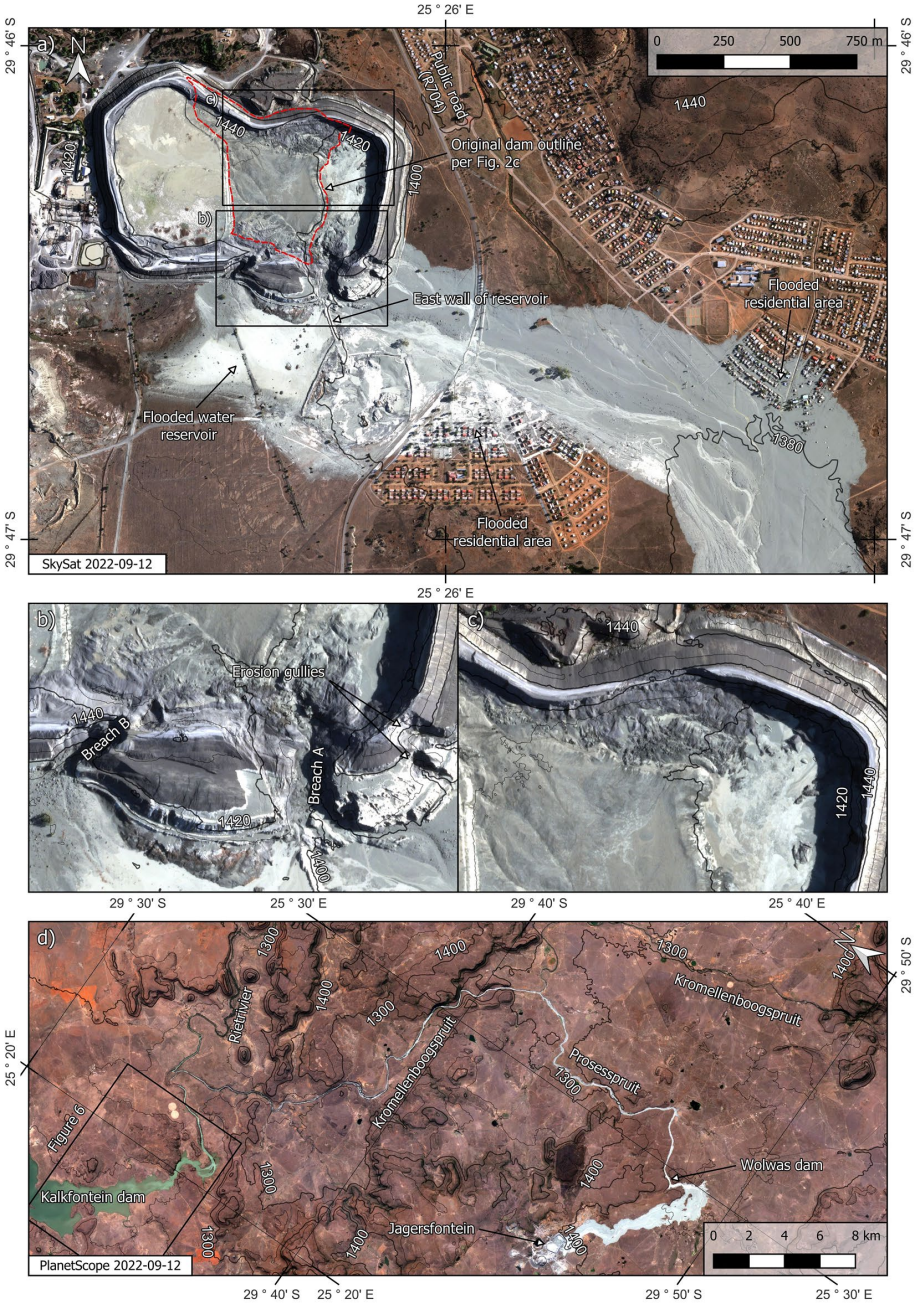
The post-failure scenario. (**a**) Impact on the immediate surroundings. (**b**) Enlargement of breached area. (**c**) Enlargement of collapsed northern wall. (**d**) Full extent of the tailings runout. NB: see also Fig. S2 for expanded version of part (**a**). In part (**d**), words with the suffixes *-spruit* or *-rivier* refer to the names of rivers. NB: Contour lines indicate metres above sea level. Created using QGIS software version 3.26 (http://www.QGIS.org).

The dam failed close to its southeastern corner and also experienced instability in its north perimeter wall (Figs. 5a–c and S2). The released tailings escaped primarily from the original dam and the east expansion. Tailings in the west expansion above the internal wall may have also escaped. Assuming that the level of tailings prior to failure was between 1435 and 1440 masl, we estimate the released volume of tailings to be between 4 and 6 million m^3^. This range agrees with an estimate of 5 million m^3^ based on stereoscopic analysis of satellite images^17^. The geometry of the tailings that remained inside the dam suggests that most of the tailings deposited prior to 2010 were not mobilised by the failure (Fig. 5a). Two separate breaches A and B and two large erosion gullies developed during the failure (Fig. 5b). A supplementary file (Video S1) contains an animation that further aids an understanding of the post-failure geometry of the dam.

**Figure 6:**
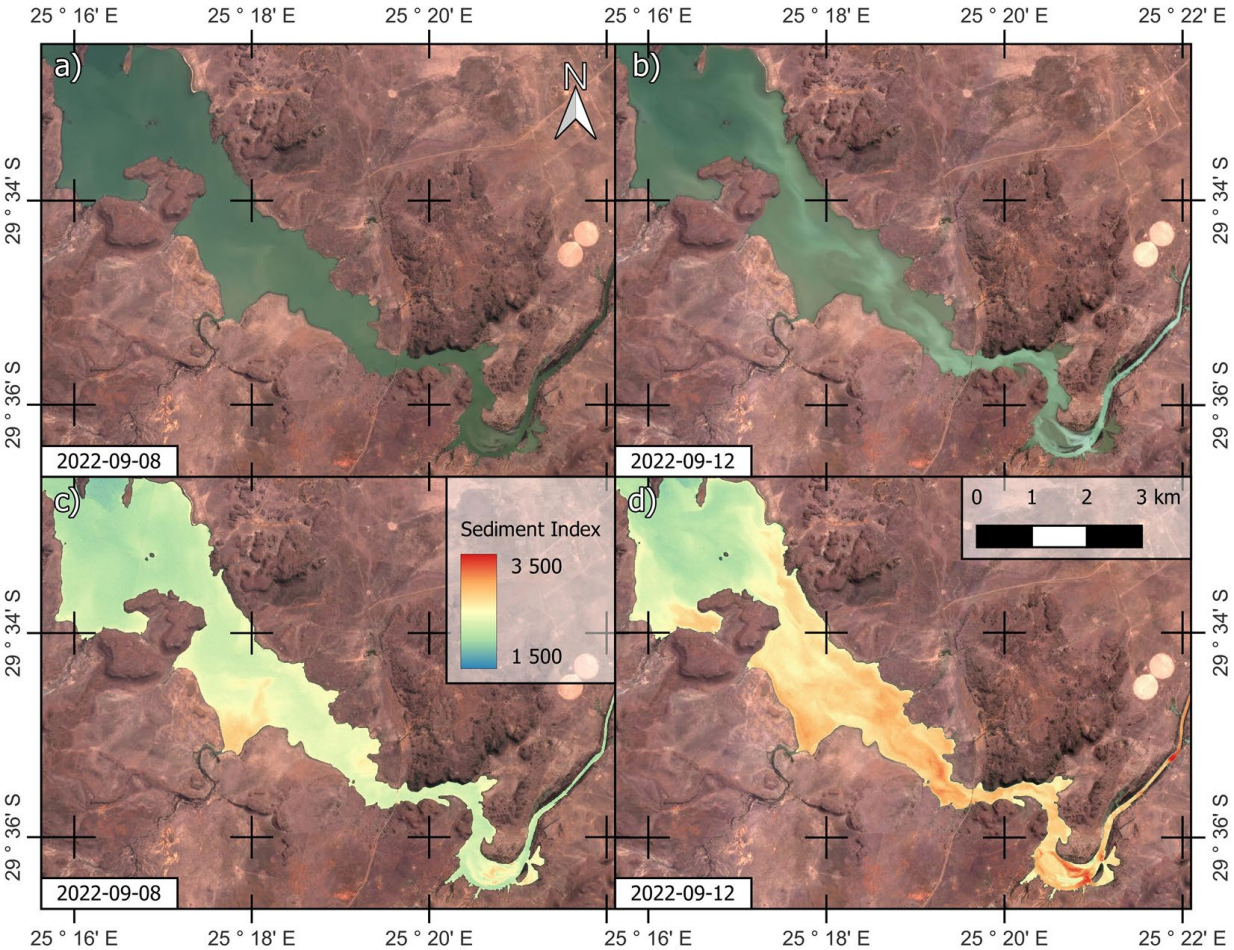
Pre and post-failure PlanetScope images of the upper part of the Kalkfontein dam. (**a**) and (**b**) Natural colour renderings. (**c**) and (**d**) Sediment index rendering. NB: Parts (**c**) and (**d**) share the same Sediment Index colour ramp. See greater context in Fig. 5d. Created using QGIS software version 3.26 (http://www.QGIS.org).

The mudflow travelled ~ 7 km over dry land before reaching the reservoir of the Wolwas dam (Fig. 5d). Tailings continued to flow along ~ 56 km of streams and rivers causing some of them to overflow and take on a pale colour. Pre and post-failure natural colour images and sediment index^19,20^ renderings strongly suggest that the tailings had reached the Kalkfontein dam reservoir by 12 September (Fig. 6).

## Discussion

The discerned construction and operation history of the dam raises several concerns. For instance, Fig. 3e showed a wet spot on the portion of the outer wall on which breach B eventually developed. This image was captured on 9 February 2021 yet weather records do not show any significant rainfall events during the five preceding days (Fig. S3a). This suggests that the wet spot in Fig. 3e was caused by impounded water seeping through the perimeter wall and then cascading down the slope creating the erosion gullies. This is consistent with the large decant pond present in the dam on the same day and the day before the wet spot was observed (Figs. 2h and 4f). Large amounts of water in a tailings dam (Fig. 4) can initiate failure mechanisms, exacerbate the consequences of failure by enhancing tailings mobility, or both^9,10,21^. Furthermore, the supernatant pond was on occasions located against the perimeter wall (Fig. 4), whereas recommended practice in dams built progressively is to keep a relatively small pond near the centre of the impoundment. This promotes safety by enabling the development of long beaches between the pond and the perimeter wall^9^ which is particularly important when dams do not have distinct core and filter zones. However, even in zoned dams, the absence of beaches can contribute to exacerbate failure consequences^21^. Lastly, tailings deposition predominantly from the west end of the dam (Figs. 2, 3 and 4) contravenes best practice which calls for deposition to proceed sequentially from different points on the perimeter wall. This allows greater control over the position of the decant pond and enables tailings deposited in one area to dry and strengthen while deposition proceeds in a different area^9,10^.

Considering the geometry of the breached wall (Fig. 5b), our hypothesized general failure sequence is as follows. Breach B developed first, possibly due to a runaway erosion gully that began as shown in Fig. 3e. The fact that the wet spot in Fig. 3e coincides with the location of breach B supports this possibility. Furthermore, the absence of heavy rainfall events in the weeks prior to failure (Fig. S3b), coupled with the absence of a decant pond only five days before failure (Fig. 4h), suggests that it is unlikely that failure was triggered by an overtopping event. Similarly, there have been no reports of seismic events preceding the failure, suggesting that it was not triggered by an earthquake. From the images presented herein, the postulated runaway erosion mechanism (Fig. 3e) thus emerges as the most likely triggering event. The tailings released through breach B flooded the adjacent water reservoir causing the erosion of its east wall which lies at the toe of breach A (Fig. 5a). Loss of toe support then triggered further instability leading to breach A. Slurry flowing along the benches of the dam that are close to breach A subsequently led to the development of the large erosion gullies (Fig. 5b). This last observation is based on helicopter footage captured on the day of the failure^22^.

The preceding hypothetical failure sequence leads to the question of whether the initiating internal seepage event could have occurred in the apparent absence of a large decant pond during the months prior to failure (Fig. 4h). We believe that this is indeed possible because the low permeability of tailings resulting from diamond mining can enable them to retain water for extended periods of time^9^. Perhaps more importantly, video footage of the failure (e.g.^22^) and the mobility of the mudflow (Fig. 5) both indicate that, even if water was not ponded atop the dam, there were large amounts of interstitial water in the dam at the time of the failure. Notwithstanding, we acknowledge that validation of the proposed failure sequence requires an in situ geotechnical investigation.

It is pertinent to also highlight the limitations of the approach adopted herein. For instance, temporal and spatial resolutions are key considerations. The temporal resolution determines how often images are acquired. The Sentinel-2 satellite mission has a nominal revisit time of five days^23^. The prevalence of clouds can further increase the time interval between useful Sentinel-2 images. But even a temporal resolution of five days implies that while the data can be used to assess adherence to some aspects of good engineering practices, it clearly cannot be used to monitor against brittle failure mechanisms that develop over minutes or seconds (e.g.^14,21^). The temporal resolution of the images obtained herein from the South African Chief Directorate of National Geo-Spatial Information (NGI) and Google Earth Pro is even more limited and there can often be months or years between consecutive images from these sources.

As for the spatial resolution, it controls the level of detail that can be discerned from the images. The Sentinel-2 satellites collect visible and near infrared light at a resolution of 10 m/pixel^23^. Only relatively coarse aspects of a tailings dam operation can be discerned at this resolution. As illustrated herein, examples include changes to the layout of the dam, broad deposition patterns and extent of the decant pond. Images from Google Earth Pro and the NGI have higher spatial resolutions that can reveal smaller details such as furrows, seepage, and location of construction equipment and of other mine infrastructure. However, one disadvantage of these last two sources is that they do not provide a near infrared band which is useful in delineating the decant pond of tailings dams^12,24^. Resort to commercial remotely sensed data can overcome some of the limitations in temporal and spatial resolution of publicly available data.

To provide a more complete context around the failure, it is useful to briefly touch on some aspects which fall beyond the scope of our paper. For instance, analysis of radar satellite data indicates that the portion of the wall that failed may have been bulging outward during the month preceding the failure^16^. An aquifer underlying the tailings dam has also been flagged as an important contributing factor^8^.

Other factors that we have not addressed go beyond the physical failure mechanism. These include regulatory and organisational factors which also play an important role in these tragic events^25^. For example, since at least 2012, Jagersfontein Developments unsuccessfully applied for permission to deposit tailings in the open pit, visible in Fig. 1^26,27^. The permit was ultimately issued a few days after the failure as a means of mitigating the danger posed by the remnants of the dam^28^. Additionally, our introduction noted an ownership change a few months before failure. Such changes pose challenges to ensuring continuity of good construction practices for tailings dams^29^. Also, reports in the media indicate that high water levels in the dam led South African authorities to order a shutdown in 2020 which lasted until June 2021^30^. Other reports suggest that the Jagersfontein dam may have fallen in a regulatory loophole as it was not under the oversight of the South African Department of Mineral Resources and Energy. The reasons for this relate to the fact that operations focused on the reprocessing of legacy mine waste as opposed to extraction of ore^31^.

As has been the case following other dam failures, the work of an officially appointed and independent investigation team will be critical in understanding how these and other issues may have combined to create this catastrophe^13,14,21^. The findings of such investigation efforts help the tailings industry move closer to fulfilling the "zero harm" aspiration of the Global Industry Standard for Tailings Management^5^.

## Concluding remarks

We used publicly available remotely sensed data to investigate the construction and operation history of the Jagersfontein dam and identify deviations from best practice. It is worth highlighting that we used the Google Earth Pro and the EO Browser platforms to access, screen or analyse some of the satellite images (see “Methods” section). These two platforms are free for non-commercial purposes and have intuitive interfaces that require minimal training. Notwithstanding, and as shown herein, when coupled with an understanding of the behaviour of tailings dams these platforms constitute useful starting points to empower stakeholders interested in proactively monitoring these structures. This is particularly relevant in jurisdictions with limited resources in which the vulnerability of communities may exacerbate the consequences of failure^32^. Furthermore, some investigations in the field of surveillance cameras show that people exhibit better adherence to rules when they know they are being observed^33^. We posit that greater awareness of the capabilities of publicly available remotely sensed data can encourage adherence to good construction and operation practices. Considering the significant uncertainties that remain in methods commonly used to assess the stability of tailings dams^10^, a robust monitoring strategy should focus on their construction and operation^34^. That is, it is relatively easy to determine whether dam construction is following best practices, yet difficult to determine whether an improperly constructed dam is about to fail.

## Methods

The investigation required collecting optical remotely sensed data to assess (a) the construction history of the dam and (b) the consequences of failure. We used only publicly available data to investigate the construction history. In particular, our focus was on the identification of departure from good construction practices that can contribute to failure. Accordingly, we sought relatively high resolution images, preferably ≤ 10 m/pixel, which enabled the identification of aspects relevant to geotechnical stability. These aspects include the extent and location of the decant pond, tailings deposition patterns, integrity of the retaining wall, and seepage of water through the wall^9,10^. Additionally, we collected rainfall data to provide context to the wet spot in Fig. 3e and assess the likelihood of the failure being triggered by rain. Assessment of the consequences of failure relied on commercial satellite imagery. In particular, to assess the post-failure geometry of the dam, we relied on a stereo pair image that enabled the reconstruction of a digital elevation model (DEM). The following paragraphs describe how we sourced and processed the data.

To investigate the construction sequence of the dam (Fig. 2) we used the following open access sources: (a) very high resolution (< 1 m/pixel) satellite images available via Google Earth Pro (http://www.google.com/earth/download), (b) true colour renderings of data captured by the Landsat 8 satellite (sharpened to 15 m/pixel)^35^, and (c) aerial imagery (< 0.5 m/pixel) available through the South African Chief Directorate of National Geo-Spatial Information (NGI) website (http://www.cdngiportal.co.za) and reproduced herein with permission. The images from Google Earth Pro and the NGI have much higher spatial resolutions than Landsat 8 images. However, no images from the first two sources were available for the period between May 2010 and August 2015. This results in considerable uncertainty regarding the operation of the dam during this period. To reduce this uncertainty, we used the first Landsat 8 image available for the site which was captured on 10 April 2013 (Fig. 2b). Despite its lower resolution, the Landsat 8 image allows assessment of changes in the overall layout of the dam.

We investigated the rainfall history of the site by accessing the 'hourlyPrecipRateGC' band of the 'GSMaP Operational: Global Satellite Mapping of Precipitation' dataset^36^ via Google Earth Engine (GEE)^37^. The 'hourlyPrecipRateGC' band contains satellite-based estimates of rainfall corrected by rain gauge data^38^. We cross checked the data against records from the closest functional weather station operated by the South African Weather Service (SAWS). This weather station is in Fauresmith, located ~ 10 km to the west of Jagersfontein. These records were provided by SAWS upon request.

The historical behaviour of the pond of supernatant water that accumulates atop tailings dams is critical to their stability^9,14,21^. To investigate this aspect of the Jagersfontein dam (Fig. 4), we used the normalised difference water index (NDWI) and true colour renderings^18,24^ as computed using Sentinel-2 Level 1C data atmospherically corrected to provide surface reflectance^23^. We used Eq. (1) to compute NDWI in QGIS software version 3.26 (http://www.QGIS.org) using bands 3 and 8 of Sentinel-2 images, which correspond to green and near infrared reflectance, respectively.

1 $$NDWI=\frac{Green-NIR}{Green+NIR}$$

We performed the atmospheric correction using the sensor-invariant atmospheric correction (SIAC) method^39^. We did not use Sentinel-2 Level 2A data (atmospherically corrected) because the availability of this dataset for Jagersfontein only begins on December 2018 and we used Sentinel-2 images from before that date (Fig. 4a–c). In order to maintain a uniform workflow, we also did not consider the Level 2A dataset for dates after December 2018. Identification of the decant pond relied on inspection of the natural colour render of individual satellite images, assessment of the evolution of apparent water bodies over time, and renders of the NDWI. NDWI is particularly useful for the Jagersfontein dam because the supernatant water often exhibits a greyish tone, possibly due to suspended solids, which is similar to the surrounding tailings (e.g. Fig. 1). Conversely, the high values of NDWI that are characteristic of water facilitate identification of the supernatant pond. Notwithstanding, the lack of access to the site implies that some uncertainties may remain when relying on remotely sensed data to identify free water bodies.

We used the EO Browser platform (https://apps.sentinel-hub.com/) to do initial screenings of Landsat 8 and Sentinel-2 images. We used GEE to further process images of interest. In particular, we used GEE to: (1) Access the Landsat 8, Collection 2, Tier 1 top-of-atmosphere reflectance dataset. Coverage for Jagersfontein begins in April 2013. Previous Landsat missions did not include useful images of the site and were thus not used. (2) Access the Sentinel-2 Level 1C dataset (orthorectified top-of-atmosphere reflectance). Coverage for Jagersfontein begins in September 2015. (3) Implement panchromatic sharpening to 15 m/pixel of the Landsat 8 red, green, and blue bands. (4) Produce true colour renderings of Landsat 8 and Sentinel-2 images. (5) Implement the SIAC algorithm on the Sentinel-2 Level 1C data. (6) Access the ‘SRTM Digital Elevation Data Version 4’ dataset which provides a global DEM based on the Shuttle Radar Topography Mission (SRTM)^40^.

On the day of the failure of the Jagersfontein dam (i.e. 11 September 2022), we tasked a stereo pair image of the site using the SkySat satellite constellation of Planet Labs (http://www.planet.com). The stereo pair image was acquired on 12 September at 08:04 local time by the satellite SSC16. The image presented in Figs. 5a and S2 has been orthorectified and panchromatically sharpened to 0.5 m/pixel. Both images were acquired with a sun azimuth and sun elevation angle of 72° and 21°, respectively. The view angle of the first and second image were 17.0° and 15.3°, respectively. The satellite azimuth angle of the first and second image were 341.6° and 116.0°, respectively.

We computed a digital elevation model DEM from the stereo pair using the Agisoft Metashape Professional software version 1.8.4 (http://www.agisoft.com). To this end we used the unrectified panchromatic SkySat Scene of the stereo pair imagery as supplied by Planet Labs. The rational polynomial coefficient files required by the software to perform the orthorectification and photogrammetric calculations were used as supplied by Planet Labs. We aligned the stereo pair DEM to the SRTM DEM using QGIS. The contour lines in Fig. 5a–c are based on the DEM computed from the SkySat stereo pair image whereas the contour lines for the greater area shown in Fig. 5d are based on the SRTM DEM. We also used the QGIS software to generate an animation that illustrates the post-failure geometry of the dam and which is presented as a supplementary file (Video S1).

Our estimations of released impounded tailings assumed a constant level of tailings in the dam prior to the failure. Based on the post-failure geometry and imagery, we estimated that the pre-failure level of tailings was between 1435 and 1440 masl. The released volume of tailings was then computed as the volume above the DEM but below the assumed pre-failure level of tailings. The calculation domain was confined to the area of the dam within the perimeter wall.

To investigate the full extent of the tailings runout we accessed the PlanetScope catalogue of Planet Labs (http://www.planet.com). PlanetScope images have a spatial resolution of ~ 3 m/pixel. We inspected pre and post-failure images captured on 8 and 12 September 2022, respectively, to assess the reach of the tailings runout (Fig. 5d). Besides inspecting natural colour renderings of these images (Fig. 6a,b), we also inspected renderings of the sediment index (SI) as defined in Eq. (2)^19^ to further assess whether tailings had reached the Kalkfontein dam (Fig. 6c,d). The sediment index has been previously used to assess the pollution of fresh water bodies caused by a tailings dam failure^20^.

2 $$SI=\frac{Green+Red}{Green/Red}$$

## Supplementary Information

Supplementary Information.

Supplementary Video S1.

## Data Availability

The GEE code and stereo pair DEM used and/or analysed during the current study are available from the corresponding author on reasonable request. The raw stereo pair images of the Jagersfontein dam failure are available from Planet Labs (http://www.planet.com).
