# Supplementary material for: Public remotely sensed data raise concerns about history of failed Jagersfontein dam
Source: Sci Rep. 2023 Apr 5;13:4953. doi: 10.1038/s41598-023-31633-5 (PMC10076419; doi:10.1038/s41598-023-31633-5)
Supplement: Supplementary file 1 — Supplementary Information. [file 41598_2023_31633_MOESM1_ESM.docx]

**Supplementary Figures**


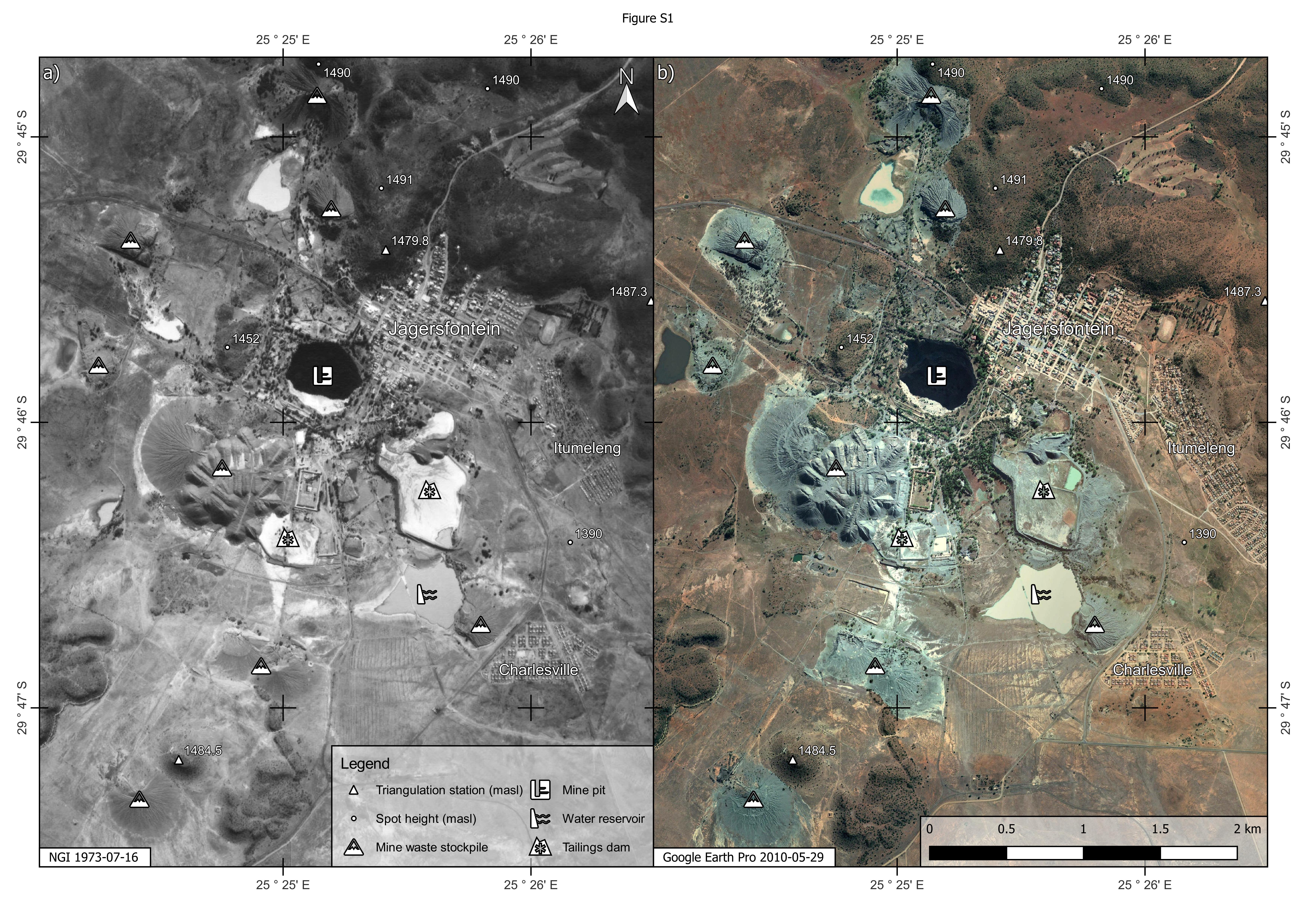


**Figure S1.** The Jagersfontein dam and its immediate surroundings. **a,** Aerial image from 1973 (two years after ore extraction ceased). **b,** Satellite image from 2010. Maps created using QGIS software version 3.26 ([www.QGIS.org](http://www.QGIS.org)).


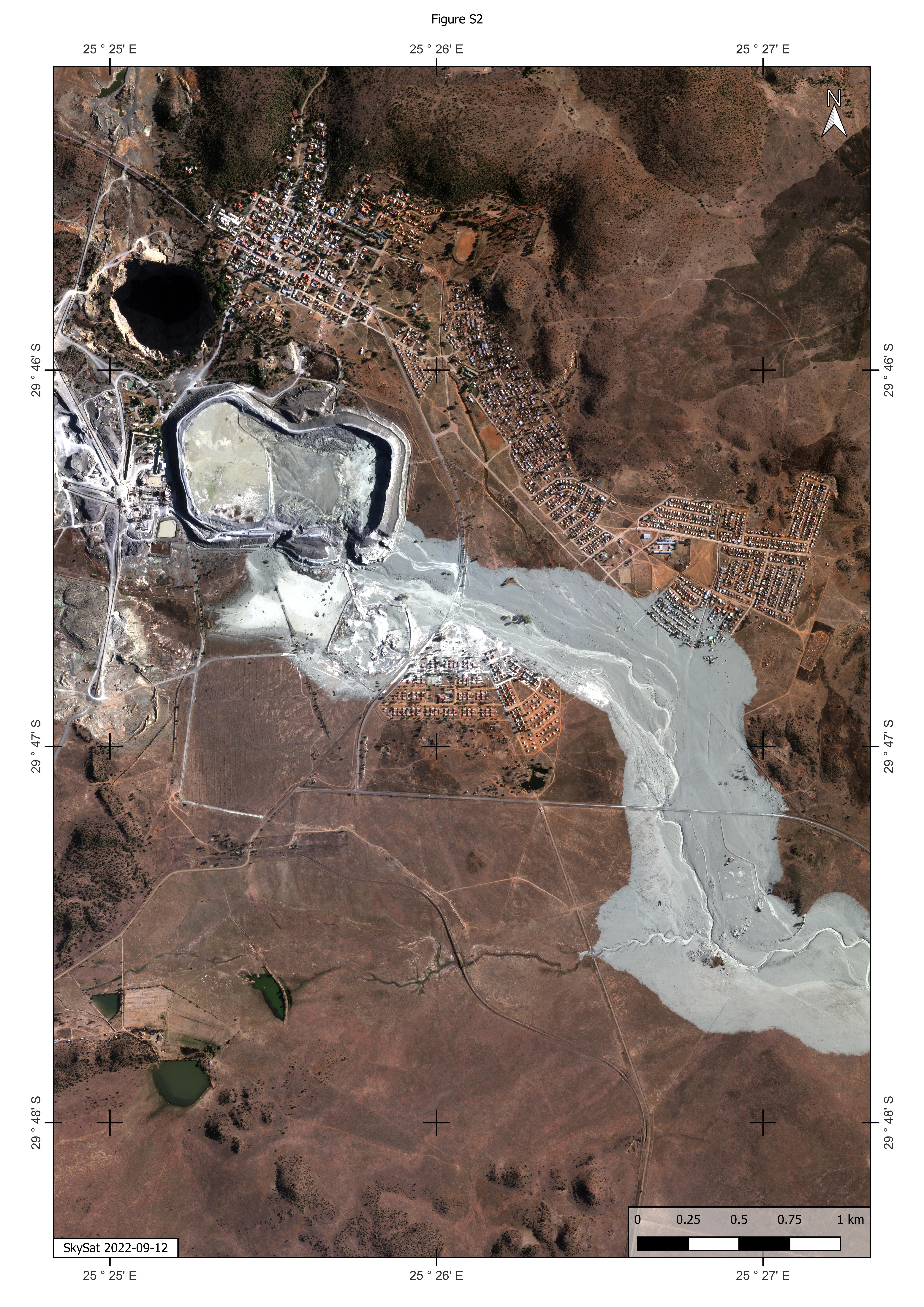


**Figure S2.** Tailings runout from the Jagersfontein dam a day after the failure. Map created using QGIS software version 3.26 ([www.QGIS.org](http://www.QGIS.org)).

**Figure S3.** Rainfall data at Jagersfontein dam. **a,** Weeks leading up to the observation of the wet spot depicted in Fig. 3e. **b,** Weeks leading up to the failure of the dam on 11 September 2022.

**Video S1.** Animation of the Jagersfontein dam post failure digital elevation model obtained from SkySat stereo pair imagery acquired on 12 September 2022. Video rendered using QGIS software version 3.26 ([www.QGIS.org](http://www.QGIS.org)).
